# The Impact of Physical Education Based on the Adventure Education Programme on Self-Esteem and Social Competences of Adolescent Boys

**DOI:** 10.3390/ijerph18063021

**Published:** 2021-03-15

**Authors:** Agnieszka Koszałka-Silska, Agata Korcz, Agata Wiza

**Affiliations:** 1 Department of Pedagogy, Poznan University of Physical Education, Królowej Jadwigi 27/39, 61-871 Poznan, Poland; wiza@awf.poznan.pl; 2 Department of Didactics of Physical Activity, Poznan University of Physical Education, Królowej Jadwigi 27/39, 61-871 Poznan, Poland; korcz@awf.poznan.pl

**Keywords:** social competences, self-esteem, physical education, physical activity, school-based intervention, adolescents, adventure education

## Abstract

The objective of this study is to analyse the impact of physical education based on the adventure education programme on the social competences of adolescent boys. The participants (n = 70) were 1st grade high school students between 15 and 16 years old. Adolescents’ social competences were measured using the Rosenberg’s Self-Esteem Scale (RSES) and Social Competence Questionnaire (SCQ) before and after the intervention. An experimental repeated-measures design was used, with a comparison group. ANOVA (2 × 2) for interaction group x time showed statistical significance in competences revealed in situations of social exposure (F_1, 68_ = 5.16, *p* < 0.05, partial η^2^ = 0.07) and competences revealed in situations requiring assertiveness (F_1, 68_ = 4.73, *p* < 0.05, partial η^2^ = 0.07). Using the adventure education (AE) programme may be recommended as a way of developing social skill competences revealed in situations of social exposure and competences revealed in situations requiring the assertiveness of adolescents through physical activity that can be easily integrated into the school environment.

## 1. Introduction

Researchers broadly agree that there is a need to develop young people’s social competences and that their positive self-esteem plays a very important factor in their lives [1,2,3]. This assumption implies the need to create conditions which will facilitate the development of social competences and self-esteem in institutional teaching and education. Both social competences and self-esteem are perceived as factors that determine one’s mental health, as well as positive functioning during adolescence [2,4,5,6] p. 7. In this context, a short transition period, such as attending a high school, is of great importance [7]. Adolescence is the time when one’s self-esteem, well-being, and perception of success change dynamically [8]. Researchers point to the relationship between deficiencies in developing social skills in childhood and adolescence and difficulties when reaching adulthood and having to adopt new social, family, and professional roles [9] pp. 249–251. Social skills can also be the criterion for assessing one’s labour market opportunities as labour stocks and potential candidates, while their deficiency can lead to the marginalisation of individuals or social exclusion [2,10]. Social competences in the scientific literature and guidelines regarding work in education and care are considered the key objectives of education in the 21st century [3]. They are perceived as an individual’s resources, which significantly determine the satisfaction derived from different spheres of life, such as health, work, or interpersonal relationships [11].

Social competences necessary in adult life and professional work develop significantly during early and late adolescence [9] p. 250; consequently, school seems to be the most appropriate place for activities aimed at acquiring social competences and positive self-esteem. 

Matczak [6] p. 7 defines social competences as complex skills that determine one’s efficiency when dealing with particular types of social settings, acquired by an individual in the course of social training. They can be developed intentionally through participation in training, whose effectiveness depends on social and emotional intelligence, temperament and personality traits, the impact of the environment on the individual, the intentional and unintentional influences of the teachers and counsellors, training and therapeutic influences [6].

The students with low levels of social competences are more likely to be rejected by their peers, which can cause reduced interpersonal contacts as well as a lack of opportunities for developing these competences [12,13], and consequently, also lower self-esteem [14,15]. Many studies have noted that adolescents with low self-esteem prefer solitude, which is probably because of the social and emotional disorders in their development [15,16,17,18]. People with higher self-esteem are braver when establishing new relationships and are more willing to do so when compared to individuals with lower levels of this trait [12]. The level of self-esteem is defined as the result of one’s real social acceptance or real social exclusion or rejection [5]. Adolescents with low self-esteem are not easily accepted by their classmates in school, and they are more likely to be separated from their peers and spend more time alone [19,20]. The efficiency of functioning in different social situations is associated with self-esteem [5]. 

Adolescents’ self-esteem is related to social functioning [15,21,22]. When developing the self-assessment tool for measuring one’s self-esteem, Rosenberg defined it as “a positive or negative attitude toward self, a type of global self-assessment. A higher level of self-esteem means the belief that the individual is good enough and worthy. Analogically, lower self-esteem means dissatisfaction with one’s self and rejecting it” [23] pp. 30–31. Self-esteem is an important part of an individual’s self-concept and is considered important for positive mental health and functioning during adolescence [4,7]. Some mental health problems (including depression, anxiety, and behavioural problems that tend to be associated with them) occur in people with low self-esteem [4,5,7]. Self-esteem is interesting both as a factor related to symptoms of depression and as an important resource for mental well-being during adolescence [4,7]. Individuals with high self-esteem may also be more likely to identify and use different personal and contextual coping resources (e.g., seek and receive more social support), which may, in turn, facilitate positive coping behaviours and adjustment, and promote well-being [4]. 

Self-esteem levels also seem to be related to various health behaviours, such as doing sports, physical activity levels, eating disorders, or addiction tendencies [24,25,26,27,28]. According to Lee, Beak, and Nicholson [24], a greater level of self-esteem significantly decreases the drinking behaviour of young people. Nemček, Kraček, and Peráčková [25] analysed differences in the self-esteem levels of elite and competitive athletes, recreational athletes, and inactive individuals, and found that physically active groups had significantly higher self-esteem. Russo et al. found that physical activity (PA) affects self-esteem, and specifically showed that adolescents with higher fitness skills have higher self-esteem compared to people in poorer physical shape [26]. Self-esteem is integral to an adolescent’s sense of their own value, a principal component of mental health, a powerful indicator of a healthy lifestyle, and an important indicator of well-being. Self-esteem is a major factor influencing adolescent lives [29]. 

Both the social skills of young people and their self-esteem are an important part of personal development and can also be developed through PA. Furthermore, social competence is frequently recognised as a curricular goal in several programmes and subjects, such as physical education (PE) [3,28]. Evidence suggests that young people can develop social skills through participation in PA [30]. Hardman et al. [31] noted that personal and social development constitutes one of the main and most frequently cited goals of European PE programmes [31]. The Polish PE curriculum includes goals for developing the social and personal competences of students at both primary and high schools [32]. Therefore, both researchers and practitioners are increasingly interested in the role of PE in preparing young people for their everyday life [33] p. 116. 

The development of social competences through PA is closely related to Adventure Education (AE) [34]. Asensio-Ramon et al. [35] emphasize that AE promotes the development of learning resources for life in adult society. Priest and Gass [36] p. 29 defined AE as “a branch of outdoor education concerned primarily with interpersonal and intrapersonal relationships. It uses adventurous activities that provide a group or an individual with compelling tasks to accomplish. These tasks often involve group problem solving (requiring decision-making, judgment, cooperation, communication, and trust) and personal challenge (testing competence against mental, social, or physical risks)” [36] p. 29. To maximize safety, professionals structure risk in a method that causes people to perceive it as enormously high, thus it is much lower and more acceptable as a medium for driving development and change. By responding to apparently insurmountable tasks, people often learn to overcome their self-imposed perceptions of their capabilities to succeed. They turn limitations into abilities, and therefore they learn a great deal about themselves and how they relate to others [31] p. 29. AE develops many different skills. The most well-known meta-analysis of research on AE identified the growth of competence in the following six categories: leadership, self-concept, academic, personality, interpersonal relationships, being adventurous [37]. There is robust research evidence on the positive effects of AE on social competences [35,37,38] pp. 158–161. The well-documented effectiveness of AE has led many countries to introduce outdoor and AE into their national PE curricula [34,39].

In Poland, the concept of AE is still relatively new. However, several researchers appreciated the potential of experimental pedagogy lessons and recommended including them in school subjects, especially in classes of PE [40,41].

This study developed, applied, and evaluated an AE programme to improve students’ social skills as well as their self-esteem. The programme was prepared following the core principles of AE and pursued the objectives of the PE curriculum at the specific educational stage. The study examined whether an AE programme would benefit the students’ social skills or their self-esteem. The intervention was introduced to classes where girls and boys learn together; however, only the boys’ results were considered. Gender differentiates the level of social competences [6] pp. 50–51 and self-esteem [5,7,37] of adolescents; therefore, the boys’ results were separated and analysed. Furthermore, it was not possible to analyse the girls’ results because this group was too small to perform statistical analyses (only five girls in the experimental group).

## 2. Materials and Methods

### 2.1. Participants

The study group comprises 70 boys aged between 15 and 16 years old (mean = 15.8 ± 0.4), who were students of the first classes of a technical high school in Poznan (a five-year post-primary school whose aim is to prepare its students to begin professional work and/or continue education). Four classes were included in the study, two of which were in the experimental group and the other two in the control group. The experimental group consists of 40 boys and the control group comprises 30 boys. Only the students whose parents gave written consent to their children’s participation in the study took part in the AE programme. Consent was also obtained for publishing the research results. The full description and purpose of the research were presented to parents and adolescents. Participants took part voluntarily and signed informed consent forms. Only a complete set of data was used for the statistical analyses. The questionnaires were completed in whole-class groups during one PE class in quiet conditions and took approximately 40 min to complete. The general characteristics of the participants at the pre-test are found in Table 1.

In the total sample of 70 students, the moderate and vigorous physical activity (MVPA) indicator averaged M = 3.7 ± 0.4. The results obtained in both the experimental group M = 3.4 ± 1.4 and the control group M = 4.2 ± 1.6 are below the recommended MVPA level of adolescents [42]. 

In the analysis of the family structure, it was shown that none of the participants lived in an orphanage or foster family. Most students from the total sample of n = 70 assessed the wealth of their family as average (39 people), 26 students assessed their family as rather wealthy, 3 people assessed their families as wealthy, and 2 people assessed their families as rather poor. No one judged his family as poor. The vast majority of students in the total group indicated their mother’s higher education (50 people). Eighteen people out of 70 indicated their mother’s secondary education, and 2 people indicated primary education. Father’s higher education was indicated by 42 students. Twenty-seven people indicated that their father has secondary education and only 1 adolescent indicated primary education.

### 2.2. Procedure and Structure of Intervention Programme

The experimental group took part in an intervention programme during PE classes. They completed a specially designed AE programme aimed at developing the students’ social competences. The control group took part in PE classes carried out in concordance with the existing curriculum prepared by the PE teaching staff/with the school’s adopted PE curriculum.

The AE programme was carried out during the classes of PE and lasted 12 weeks. The time of the intervention was selected based on recommendations from research on AE-based interventions that develop youth social competences [38]. Research from these studies recommended shortening the intervention programmes below 20 weeks to maintain a high level of motivation of participants [38]. Furthermore, the intervention was limited to 12 weeks because it had to fit into a school phase, which was not interrupted by winter break to control confounding effects of the holiday activities. The study was conducted in the first semester of the school year 2019/2020. The intervention programme was held once a week during two joined teaching units (2 × 45 min). It has been designed to fulfil the objectives of the PE curriculum at the fourth educational stage, with particular emphasis on the social competence of the students. The focus of the intervention was on improving the students’ assertiveness, social exposure, and the skills necessary for effective behaviours in close interpersonal relationships and increasing the students’ self-esteem. The innovative nature of the intervention programme comprised conducting the classes under the principles of AE and introducing during the classes of PE the elements characteristic of AE, such as actions and interactions, reflection, and transfer, all of which enable learning through real, first-hand experience. The intervention programme was based on the assumption that developing social competences can occur in social training [6] p. 7; therefore, the classes were purely practical and grounded on a variety of different physical activities. Depending on the weather conditions, the classes were held on the school field, the school gym, or a classroom. The overview of the programme has been added in the Supplementary Material as Table S1. In the Supplementary Material, it is shown how adventure education is related to the tasks based on it, their structure/course and particular objectives. The course always consisted of three basic stages:

1. A warm-up in the form of a dynamic game.
2. The main part containing problem-solving tasks that need collaboration, group actions and interactions, and trust games.
3. Reflection and transfer in the form of the students’ presentation of their conclusions, drawn from their experience and with various forms of expression, such as speaking in front of other students, writing the conclusions on pieces of paper, writing letters to one another, expressing publicly the wishes and expectations for each other as well as the whole group, or summarising the classes in one, most relevant word.

### 2.3. Ethics

The study was conducted under the Declaration of Helsinki, and the protocol was approved by the Local Bioethics Committee of the Medical University of Karol Marcinkowski in Poznan (decision no. 467/19). For the students’ convenience, the information about the anonymous and voluntary nature of their participation was read out before completing the questionnaire, that the study records would be kept confidential, and that their individual contributions would be unidentifiable in the final report. Written consent was collected from the parents.

### 2.4. Instruments

#### 2.4.1. Rosenberg’s Self-Esteem Scale (RSES)

Self-esteem was measured using the Polish version of the Rosenberg’s Self-Esteem Scale [5]. The scale is a one-dimensional tool that allows the assessment of the level of global self-esteem. The scale consists of 10 items scaled on a four-point response structure (1 = strongly disagree to 4 = strongly agree). The range of possible results is from 10 to 40 points. To assess whether the score obtained is low or high, reference should be made to the sten norms [43] p. 63. 

When scoring on this scale, the following interpretation of the results was adopted: marks 1–3 are low scores, marks 4–7 are average scores, and marks 8–10 are high scores. Five items are positively worded and five items are negatively worded to inhibit response bias, that is, an individual’s tendency to agree with statements regardless of their content. A sample item is: “On the whole, I am satisfied with myself”. In the way the answers are assessed, the positive statements are reversed: 1,2,4,6,7 so that a higher point value is awarded for answers expressing a higher self-esteem level. The Rosenberg scale has been widely used for evaluating the self-esteem of young people, and its reliability and validity are well documented. The Polish version of the scale is a reliable tool, Alpha Cronbach = 0.81–0.83, with confirmed theoretical validity [5]. Alpha Cronbach calculated on the present whole sample in pre-test was 0.89 (in post-test it was 0.92).

#### 2.4.2. Social Competence Questionnaire Version for Adolescents (SCQ)

The study used a Polish questionnaire designed to study the social competences of adolescents [6]. The Social Competence Questionnaire (SCQ) is used to evaluate social competences understood as acquired skills that condition the effectiveness of human functioning in various social situations. Besides the general index, the questionnaire also provides three detailed indicators that determine the level of competences revealed in situations of social exposure (SCQ E), situations requiring assertiveness (SCQ A), and situations of close interpersonal contact (SCQ I). The SCQ for Adolescents consists of 3 scales, with each scale reaching a different score range. The SCQ I scale contains 15 items, and the scoring range is from 15 to 60. The SCQ E scale includes 18 items, and the scoring range is from 18 to 72. The SCQ A scale includes 17 items, and the score range is from 17 to 68. The respondent assesses the efficiency with which he performs them on a four-point scale described by adjectives: definitely good, not bad, rather poor, definitely bad. Raw scores (points) are assessed by relating them to the sten norms. The following categorisation is used in the evaluation of the standard scores: stens 1–3 are low scores, stens 4–7 are average scores, and stens 8–10 are high scores [6] pp. 61–62. The reliability of the SCQ measured by the concordance (Alpha Cronbach) is 0.93–0.95 [6] p. 5. Alpha Cronbach calculated on the present whole sample for the whole scale and each subscale in the pre-test was 0.81–0.95 (in post-test it was 0.85–0.96).

#### 2.4.3. The Health Behaviour in School Aged Children (HBSC)

Only selected questions were used from the Health Behaviour in School Aged Children (HBSC) Questionnaire to investigate levels of PA and chosen socioeconomic data. PA level was determined based on the MVPA index [44]. This measure corresponds to the average number of days per week with at least 60 min spent undertaking various forms of PA during which, in the participants’ subjective opinion, their heart rate increased, and they experienced a feeling of shortness of breath (higher breathing frequency). This question was adapted by HBSC from a screening test by Prochaska et al. [44] Participants were asked to answer two questions: P1: Over the past 7 days, on how many days were you physically active for a total of at least 60 min per day? P2: Over a typical or usual week, on how many days are you physically active for a total of at least 60 min per day? The MVPA index was calculated based on the following formula: MVPA = (P1 + P2)/2 where: MVPA = PA index; P1 = number of physically active days during the past 7 days; P2 = number of physically active days during a typical week. 

Furthermore, there were some questions about the adolescent’s age, family structure, family wealth, and parents’ education [45]. The above information was used during the study to get to know the participants better and describe them.

### 2.5. Data Collection and Analysis

A two-group experimental and control pre- and post-test design was adopted. All testing was completed anonymously using a code designed to match students’ responses at pre- and post-intervention without revealing the student’s identity.

The questionnaires used for statistical analysis were taken from the boys who were present at both tests’ dates: during the pre-test (conducted immediately before the beginning of the programme) and the post-test (carried out directly after the programme ended). Of the 85 participants at the pre-test, 70 completed the post-test evaluation, giving an attrition rate of 18% (=15 of 85). There was data loss due to the sickness of the participants. The response set for students was 82%.

### 2.6. Statistical Analysis

All analyses in Statistica version 13.0 (StatSoft Polska Sp. z o.o. 2020). Descriptive quantitative data are presented as mean and standard deviation (M ± SD). The normality of each variable was tested using the Shapiro–Wilk method. Qualitative variables are presented by percentage and count (%, n). In order to examine changes between the groups (experimental, control) and time (pre-test, post-test) and their changes with variables depending on self-esteem (RSES) and social competences (SCQ I, SCQ A, SCQ E) four separate two-way repeated measures analyses of variance ANOVA (2 × 2) have been conducted.F-factors (F), indexed degrees of freedom, *p* (*p*) value, and effect sizes (partial η^2^) were reported for each ANOVA.

A sign test was used to examine changes between terms (pre-test, post-test) for the variables self-esteem and social competences included as a score calculated based on a standard sten scale. The sign test is a nonparametric alternative to the two-sample independent t test. The tested variables (self-esteem and social competences) converted to standardized sten scores are of continuous distribution meeting the test’s assumptions. The sign test involves calculating a number that indicates how many times the pre-test value is higher than the post-test variable value. Cohen’s d (d) effect size was reported for significant difference. Cohen’s d-value of less than 0.2 was negligible, between 0.2 and 0.49 was small, between 0.50 and 0.8 was an admission, greater than 0.8 was a drop. The level of statistical significance was 0.05 for all statistical procedures.

## 3. Results

A separate two-way ANOVA with repeated measures (group × time) for RSES showed that there was no statistically significant main effect for group (F_1, 68_ = 2.27, *p* = 0.1362, partial η^2^ = 0.03) and for time (F_1, 68_ = 0.52, *p* = 0.4754, partial η^2^ = 0.01) between pre-test mean values of 28.0 ± 5.81 and post-test mean values of 28.3 ± 6.6. There was no statistically significant interaction between time and group (F_1, 68_ = 0.08, *p* = 0.7749, partial η^2^ < 0.01). This was confirmed by RSES analysis according to the sten scale score, which showed no statistically significant change in either the study (*p* = 0.999) or control group (*p* = 0.289). This is presented in Table 2.

**Figure 1:**
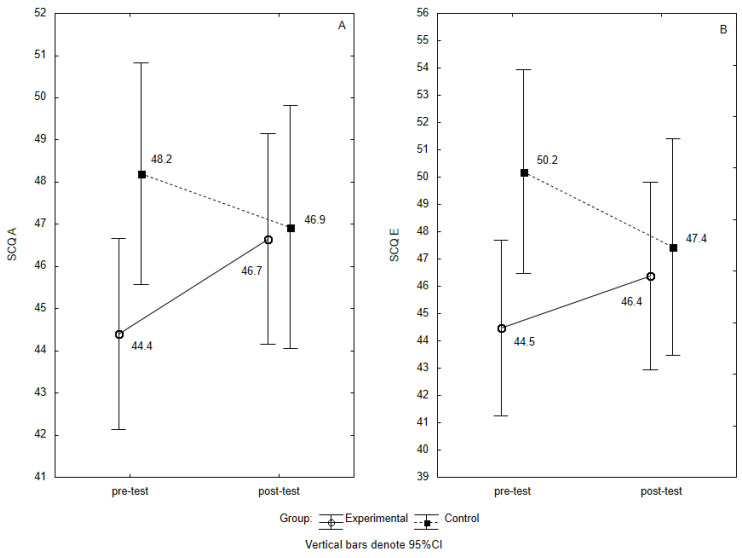
(**A**,**B**) Distribution of the SCQ A and SCQ E scale scores for the Experimental and Control groups at pre-test and post-test. The mean value for 95% confidence interval. SCQ A and SCQ E scale scores for the Experimental and Control groups at pre-test and post-test. Note: CI—confidence intervals, SCQ A—mean scores of Social Competence Questionnaire, scale A—competences revealed in situations requiring assertiveness, SCQ E—mean scores of Social Competence Questionnaire, scale E—competences revealed in situations of social exposure.

A separate two-way ANOVA with repeated measures (group × time) for SCQ I showed that there was no statistically significant main effect for group (F_1, 68_ = 3.11, *p* = 0.0824, partial η^2^ = 0.04) and for time (F_1, 68_ = 050, *p* = 0.4820, partial η^2^ = 0.01). There was no statistically significant interaction between time and group (F_1, 68_= 0.001, *p* = 0.9806, partial η^2^ < 0.01). For SCQ A, it showed that there was no statistically significant main effect for group (F_1, 58_ = 1.55, *p* = 0.2170, partial η^2^ = 0.02) and time (F_1, 68_ = 0.37, *p* = 0.5450 partial η^2^ = 0.01). In contrast, the interaction time × group showed a statistically significant difference (F_1, 68_ = 4.73, *p* = 0.0331, partial η^2^ = 0.07). This statistical variation was driven by a statistically significant increase in competences revealed in situations requiring assertiveness in the small effect size study group (post hoc: *p* = 0.0371, d = 0.30. This is presented in Figure 1A).

This was confirmed by the SCQ A analysis according to the sten scale assessment, which showed an increase in competences revealed in situations requiring assertiveness (*p*= 0.0455). In contrast, the control group in the pre-test and post-test was not statistically significantly different (*p* = 0.3035). This is presented in Table 2. For SCQ E, there was no statistically significant main effect for group (F_1, 68_ = 2.10 *p* = 0.1518, partial η^2^ = 0.03) and time (F_1, 68_ = 0.18 *p* = 0.6746, partial η^2^ < 0.01). However, the interaction time × group showed a statistically significant difference (F_1, 68_ = 5.155, *p* = 0.026, partial η^2^ = 0.07). This is presented in Figure 1B. The analysis of the SCQ E by sten scale scores showed an increase in competences revealed in situations of social exposure in the study group (*p* = 0.0159). The control group in pre-test and post-test did not differ statistically significantly (*p* = 0.9999). This is presented in Table 2.

## 4. Discussion

The purpose of the study was to evaluate the effectiveness of the AE-based programme introduced during PE classes in a high school, based on the changes in the level of social competences among boys aged 15–16. In the present study, it was hypothesized that participation in the AE programme develops some of the boys’ social skills. The first of the study’s hypotheses was partly confirmed: AE-based exercise during PE classes influenced the development of social competences related to behaviour in a situation requiring assertiveness and social competences related to behaviour in a situation of social exposure.

There has been an increase in the boys’ competences responsible for effective behaviour in situations of social exposure and situations requiring assertiveness. The competences that are responsible for effective behaviour in situations of close interpersonal contact have not changed, which can be due to several factors, including the stage of development of the group [46]. The classes that participated in the programme were newly formed groups, whose willingness to work on the skills beneficial for creating close interpersonal relationships was scarce. The newly formed group of participants who did not know each other beforehand needed more tasks integrating them and bridging the social distance, rather than assignments focused on developing skills responsible for the ability to enter close interpersonal contact, such as confiding in one another or listening to other people’s confessions. Probably due to this low degree of familiarity, the participants of the programme did not report an increase in the skills required to behave effectively in situations of close interpersonal contact. Based on a meta-analysis of the ways of achieving the assumed results of the adventure education, developed by McKenzie, several features have been cited that influence the final results [46]. It should be emphasised that the outcomes of the programme are independent of processing, which means “sorting and ordering of information” that enables the participants to internalise the meaning of the adventure education experience. The researchers also emphasise the meaning of the participants’ individual traits and personalities, and suggest that one’s personality does in fact, to some degree, determine the extent of changes that they experience during the programme [46]. An important factor in achieving the results of the programme is the size of the group. Particularly effective learning takes place in groups of 7–15 people, while a school class consists of 26 students. The inter-participant bond that binds the group affects the results of the programme [46], and in the newly formed class, the bonds between the participants were just beginning to form. Conrad and Hedin found that developing personal relationships with the other participants influenced both one’s personal and social development [47].

The second study hypothesis that participation in PE based on the AE programme increases the boys’ self-esteem was not confirmed. The study hypothesized an increase in self-esteem in students after the intervention. Nonetheless, there were no statistically significant changes in the boys’ self-esteem. Similar results were reported in an experiment examining the effectiveness of adventure education programmes as students’ expeditions into the wilderness [48]. The results of this experiment showed that the level of self-esteem in boys aged 11–18 years before and after the expedition was similar [48]. It should be noted, however, that the intervention differed significantly from the one in the present study. The mountain expedition was originally organised outside the school grounds, and only during a certain time. It lasted several days and was a onetime-only event of high intensity. It was not implemented in the weekly school activities, which were held following the school’s timetables and conditions. Another study of the participants’ self-esteem, conducted with a pre-test and a post-test, is the study on the stableness of the students’ self-esteem during the school year [7]. It demonstrates that the self-esteem of Norwegian boys at the age of 15–21 did not change during the school year [7]. The results obtained may reinforce the theoretical perspective, according to which one’s self-esteem is perceived as a relatively constant trait [5].

Self-esteem is an important part of the self-concept of an individual [4]. Hattie et al. conducted the study measuring the programme’s effect size and follow-up effect size. Of the six outcomes studied in this manner, self-concept had the least significant programme effect size, while its follow-up effect size was the most significant one [37]. This might indicate a “sleeper” effect, whereby self-concept changes are seeded during the programme and continue to grow afterwards [49].

The precisely described circumstances in which the classes took place (their duration, whereabouts, the available equipment, and the school infrastructure) determined the type of activities. The programme was based on trust games, interactions, and tasks involving communication, teamwork, and problem-solving. However, it offered no more complex activities/bigger challenges characteristic of adventure education, such as high rope activities, rock climbing, or canoeing, whose common goal is to arouse in the participants a variety of important and difficult emotions, such as fear, uncertainty, and a sense of danger [46]. Dealing with these emotions and seemingly impossible challenges allows the participants to feel much more satisfied with themselves and to prove their self-worth, and consequently increase their self-esteem. Due to the need to adapt the programme to the school infrastructure, the programme offered too few intense emotional experiences to significantly affect the participants’ self-esteem. 

The phenomenon of a participant noticing there has been a change in their behaviour is also of great importance. It is up to the individual to realise the degree to which their own perception/image of themselves has altered. The age, gender, background, and expectations of the participants may influence the outcomes they experience as a result of an adventure education programme [37,47].

The positive effects of the development of social competences through participation in adventure education programmes are well documented [33,35,36,39]. However, in the Polish educational and cultural context, regular classes of PE that include AE are innovative research.

This study complements the current literature by analysing the impact of the AE programme on students’ social competences. Social skills have been selected and identified as learning objectives, and PE lessons based on the AE programme are designed to achieve these objectives. The hypothesis has been put forward that PE based on the AE programme, compared to a traditional PE programme, will improve social skills, revealing effective behaviour in situations of social exposure and situations requiring assertiveness.

The research presented has been developed on the assumption that social skills are the goal of the PE programme and they can be achieved by including AE. These findings provide evidence that developing social skills can be an attainable goal in PE. PE teachers and youth sports coaches can improve students’ social skills by creating and implementing specially designed programmes using AE. However, it is worth noting that in the future the AE programme should be enriched with additional activities that would arouse more intense experiences among participants, and that the forms of activity should be adjusted to the individual developmental needs of students. In addition, increasing the number of classes under the programme could affect the development of other social skills of boys. However, the number of PE classes based on AE should be carefully balanced with the number of activities with a high level of physical activity (e.g., football, basketball, running). PE lessons should be strongly diversified in terms of the goals pursued and the methodology of conducting classes subordinated to them. In the presented study, the focus was on the greatest adaptation of AE classes to school conditions (duration of lessons, school infrastructure, and available teaching and sports aids), but the programme would be more if the activities were held outside the school and the school playground (in forests, lakes, parks, etc.), enabling students to have direct contact with nature and thus providing additional benefits [48].

The limitation of the study is the lack of the second post-test to check the level of the social competence and self-esteem of students in the long term. The second post-test would determine whether students have retained their acquired skills and whether their level has increased over time. As reported by Neill and Richards, the effects of AE programmes seem not only to be sustained over time but also to increase further, which is impressive [49]. The second post-test was scheduled 5 months after the end of the programme and was to take place before the end of the school year, in June 2020. Unfortunately, the planned measures were not implemented because of the coronavirus pandemic. In March 2020 (one month after the programme), a pandemic appeared in Poland and a lockdown was introduced, so students started to participate in remote teaching while still being isolated at home. It seems to be inadmissible to re-examine both self-esteem and social competences in a pandemic. Investigating the level of social competence and self-esteem in conditions of still recommended home isolation, remote learning, and permanent fear of contact with a potential COVID-19 infection would be valuable but could not be seen as a continuation of measuring the sustainability of the effects of participating in PE based on the AE programme.

The current pandemic situation associated with home isolation has a negative impact on psychological and social changes, thus exposing children to significant risks to their well-being [34]. The tested AE programme can become particularly useful for educators after the pandemic as an intervention to support the rebuilding of interpersonal relationships and to resume the process of shaping social competencies during direct social training, which has been limited or is impossible to perform. 

The data were collected at one high school in Poznan. Results can thus not be generalised to the whole Polish adolescent population.

Undoubtedly, the strength of the research is implementing the recommendations of researchers analysing the effectiveness of programmes developing the social competences of young people. The researchers emphasize the need to improve the methodology used to evaluate programmes that promote competencies in adolescents [50,51]. Therefore, methodological rigor was guaranteed in the evaluation of the programme’s effectiveness. Measurement instruments with demonstrated reliability and validity were used, and a control group was included, with pre-test and post-test measures in both groups.

A great advantage of the research is also implementing the recommendations of McKenzie [46]. She stressed the need to verify the benefits of participating in AE programmes not organised in the wilderness only. She recommends a study of the outcomes of programmes implemented in familiar environments, such as the classroom or workplace [46]. The present research is based on ten demands and has been carried out in an environment known to teenagers, i.e., in their school during their daily lessons according to the timetable. 

Since adolescents spend most of their time at school, implementing a social competence development programme in the school curriculum can be an effective way of promoting personal and social development as a foundation for teenage well-being.

PE based on AE pursues the aims and objectives of the core curriculum for teaching PE at a given educational stage; therefore, it can provide methodical support for PE teachers and other pedagogues or sports trainers. Institutions that may be interested in the results of the research are schools (especially technical and vocational, as they prepare individuals for their own entry into the labour market), youth care facilities with deficits in social competences, or facilities running rehabilitation groups. These organisations should aim to provide conditions for young people to develop emotionally, socially, and mentally.

Regarding the limitations of the study, the sample size was small, so in future research a larger sample is recommended. Additional studies in a diverse sample are still needed to replicate findings.

## 5. Conclusions

The results of this study suggest that introducing a 5-month PE based on the AE programme leads to improvements in a student’s performance social skills necessary for exhibiting assertive and social exposure behaviour. The programme does not influence the abilities related to effective behaviour patterns in close interpersonal relations. Participation in the programme does not improve the students’ self-esteem, but comparing the experimental group, it positively influences the stability of this trait, while in the control group it tends to decrease. The most important contribution to the curriculum of PE lies in a simultaneous emphasis on the importance of physical activity/AE in the process of students’ development (physical, mental, intellectual, and social) in the school environment, which is of great importance for the psycho-physical health and well-being of young people. Using the AE programme is recommended to make the lessons more attractive, so they can be introduced in school conditions, and with no specialized equipment, access to expensive materials, or special setting. Promoting the social and personal development of students is a good way to highlight the broad spectrum of benefits that come with participation in PE classes. The study contributes to the literature about the potential and the opportunities that PE classes offer for both the social and personal development of young people. The described research has important implications for PE teachers and team sports coaches, as these professional groups should strive to provide the young people they are responsible for with regular opportunities for comprehensive development, with the view to improve their well-being—both during and after they finish their educational path.

Future research on the impact of the PE lesson programme should examine the possibility of developing other social competences of adolescent boys. Equally important in subsequent studies is the performance of a post-test to examine the development of competences over time and/or to test the sustainability of the programme effects.

## Figures and Tables

**Table 1:** General characteristics of the participants (n = 70).

Variables	Experimental Group	Control Group
	n = 40	n = 30
Age (years ± SD)	15.8 ± 0.4	15.7 ± 0.5
Family structure [%(n)]		
mother	98% (39)	100% (30)
father	85% (34)	90% (27)
an orphanage or foster family/other people	0% (0)	0% (0)
Perceived family wealth [%(n)]		
wealthy	0% (0)	10% (3)
rather wealthy	35% (14)	40% (12)
average	60% (24)	50% (15)
rather poor	5% (2)	0% (0)
poor	0% (0)	0% (0)
Parental education		
Mother’s education [%(n)]		
primary education	1% (3)	1% (3)
high education	12% (30)	6% (20)
tertiary education	27% (68)	23% (77)
Father’s education [%(n)]		
primary education	1% (3)	0% (0)
high education	16% (40)	11% (37)
tertiary education	23% (58)	19% (63)
MVPA (min/day) [M ± SD]	3.4 ± 1.4	4.2 ± 1.6

Note: M = mean, SD = standard deviation, MVPA = moderate and vigorous physical activity.

**Table 2:** Descriptive statistics of the self-esteem and social competences in experimental and control groups pre- and post-intervention programme.

Variab.	Group	Pre-Test				Post-Test			
		M ± SD	Evaluation Result ^b^ Percent (n)			M ± SD	Evaluation Result Percent (n)		
			Low	Mid	High		Low	Mid	High
RSES	Experimental	27.0 ± 6.2	38% (15)	40% (16)	22% (9)	27.2 ± 7.1	40% (16)	30% (12)	30% (12)
	Control	29.0 ± 5.2	23% (7)	47% (14)	30% (9)	29.4 ± 5.9	23% (7)	33% (10)	44% (13)
SCQ I	Experimental	39.5 ± 6.4	18% (7)	70% (28)	12% (5)	40.0 ± 8.1	25% (10)	63% (25)	12% (5)
	Control	42.3 ± 7.3	17% (5)	50% (15)	33% (10)	42.7 ± 6.1	10% (3)	70% (21)	20% (6)
SCQ A	Experimental	44.4 ± 7.5 ^a^	38% (15)	60% (24)	2% (1)	46.7 ± 8.9	28% (11)	62% (25)	10% (4)
	Control	48.2 ± 6.7	17% (5)	80% (24)	3% (1)	46.9 ± 6.3	13% (4)	87% (26)	0% (0)
SCQ E	Experimental	44.5 ± 9.5	40% (16)	53% (21)	7% (3)	46.4 ± 11.2	25% (10)	60% (24)	15% (6)
	Control	50.2 ± 11.2	23% (7)	57% (17)	20% (6)	47.4 ± 10.6	17% (5)	66% (20)	17% (5)

Variab.—variables, RSES—Rosenberg Self-Esteem Scale, SCQ I—Social Competence Questionnaire, scale I—competences revealed in situations of close interpersonal contact, SCQ A—Social Competence Questionnaire, scale A—competences revealed in situations requiring assertiveness, SCQ E—Social Competence Questionnaire, scale E—competences revealed in situations of social exposure, M = mean, SD = standard deviation; Low, Mid, High—levels of self-esteem or/and social competences; ^a^ significant differences between pre-test and post-test *p* < 0.05; ^b^ significant differences between pre-test and post-test in experimental group for variables SCQ A and SCQ E *p* < 0.05.

## Data Availability

The data presented in this study are available on reasonable request from the corresponding author.

## Supplementary Materials

The following are available online at https://www.mdpi.com/1660-4601/18/6/3021/s1, Table S1: Overview of the Programme.

## References

[1] Orth U., Robins R.W. (2014). The Development of Self-Esteem. Curr. Dir. Psychol. Sci..

[2] Rosalska M. (2015). Kompetencje społeczne dorosłych w kontekście pedagogiki przeżyć. Dyskursy Młod. Andragogów.

[3] Opstoel K., Chapelle L., Prins F.J., De Meester A., Haerens L., van Tartwijk J., De Martelaer K. (2020). Personal and Social Development in Physical Education and Sports: A Review Study. Eur. Phys. Educ. Rev..

[4] Boden J.M., Fergusson D.M., Horwood L.J. (2008). Does Adolescent Self-Esteem Predict Later Life Outcomes? A Test of the Causal Role of Self-Esteem. Dev. Psychopathol..

[5] Dzwonkowska I., Lachowicz-Tabaczek K., Łaguna M. (2007). Skala samooceny SES Morrisa Rosenberga—Polska adaptacja metody. Psychol. Społeczna.

[6] Matczak A. (2012). Kwestionariusz Kompetencji Społecznych.

[7] Moksnes U.K., Reidunsdatter R.J. (2019). Self-Esteem and Mental Health in Adolescents—Level and Stability during a School Year. Nor. Epidemiol..

[8] Ahmed M.D., KingYan H., Zazed K., Niekerk R.V., Jong-Young L. The Adolescent Age Transition and the Impact of Physical Activity on Perceptions of Success, Self-Esteem and Well-Being. /paper/The-Adolescent-Age-Transition-and-the-Impact-of-on-Ahmed-KingYan/283fca5783e4f74e1bff40c4a271a5f0bf28869f.

[9] Brzezińska A., Appelt K., Ziółkowska B. (2016). Psychologia Rozwoju Człowieka.

[10] Dirks M.A., Treat T.A., Weersing V.R. (2007). Integrating Theoretical, Measurement, and Intervention Models of Youth Social Competence. Clin. Psychol. Rev..

[11] Orth U., Robins R.W., Widaman K.F. (2012). Life-Span Development of Self-Esteem and Its Effects on Important Life Outcomes. J. Pers. Soc. Psychol..

[12] Twenge J.M., Baumeister R.F., DeWall C.N., Ciarocco N.J., Bartels J.M. (2007). Social Exclusion Decreases Prosocial Behavior. J. Pers. Soc. Psychol..

[13] Meral S., Ağır M. (2019). Factors Affecting Social Exclusion, Friendship Quality, Social Competence and Emotion Management Skills and the Effect of Problem Behaviors on Related Characteristics in Adolescents. J. Educ. Train. Stud..

[14] Leary M.R. (2003). Commentary on Self-Esteem as an Interpersonal Monitor: The Sociometer Hypothesis (1995). Psychol. Inq..

[15] Baumeister R.F., Campbell J.D., Krueger J.I., Vohs K.D. (2003). Does High Self-Esteem Cause Better Performance, Interpersonal Success, Happiness, or Healthier Lifestyles?. Psychol. Sci. Public Interest J. Am. Psychol. Soc..

[16] Wang J.M., Duong M., Schwartz D., Chang L., Luo T. (2014). Interpersonal and Personal Antecedents and Consequences of Peer Victimization across Middle Childhood in Hong Kong. J. Youth Adolesc..

[17] Wang J.M. (2016). Preference-for-Solitude and Depressive Symptoms in Chinese Adolescents. Personal. Individ. Differ..

[18] Yang S.-Y., Fu S.-H., Wang P.-Y., Lin Y.-L., Lin P.-H. (2020). Are the Self-Esteem, Self-Efficacy, and Interpersonal Interaction of Junior College Students Related to the Solitude Capacity?. Int. J. Environ. Res. Public. Health.

[19] Hong F.-Y., Chiu S.-I., Huang D.-H., Chiu S.-L. (2020). Correlations Among Classroom Emotional Climate, Social Self-Efficacy, and Psychological Health of University Students in Taiwan. Educ. Urban Soc..

[20] Antonopoulou K., Chaidemenou A., Kouvava S. (2019). Peer Acceptance and Friendships among Primary School Pupils: Associations with Loneliness, Self-Esteem and School Engagement. Educ. Psychol. Pract..

[21] Ryan R.M., Deci E.L. (2000). Self-Determination Theory and the Facilitation of Intrinsic Motivation, Social Development, and Well-Being. Am. Psychol..

[22] Trzesniewski K.H., Donnellan M.B., Moffitt T.E., Robins R.W., Poulton R., Caspi A. (2006). Low Self-Esteem during Adolescence Predicts Poor Health, Criminal Behavior, and Limited Economic Prospects during Adulthood. Dev. Psychol..

[23] Rosenberg M. (1965). Society and Adolescent Self-Image.

[24] Lee S., Bael H., Nicholson A. (2020). Youth Sport Participation and Underage Drinking Behavior: The Mediating Effect of Self-Esteem. J. Phys. Educ. Sport.

[25] Nemček D. (2017). Self-Esteem Analyses in People Who Are Deaf or Hard of Hearing: A Comparison between Active and Inactive Individuals. Phys. Act. Rev..

[26] Russo G., Nigro F., Raiola G., Ceciliani A. (2019). Self-Esteem in Physically Active Middle School Students. J. Phys. Educ. Sport.

[27] Simpson C.C., Mazzeo S.E. (2017). Attitudes toward Orthorexia Nervosa Relative to DSM-5 Eating Disorders. Int. J. Eat. Disord..

[28] Lofrano-Prado M.C., Prado W.L., Barros M.V.G., de Souza S.L. (2015). Eating Disorders and Body Image Dissatisfaction among College Students. ConScientiae Saúde.

[29] Mcwhirter B.T., Besett-Alesch T.M., Horibata J., Gat I. (2002). Loneliness in High Risk Adolescents: The Role of Coping, Self-Esteem, and Empathy. J. Youth Stud..

[30] Weiss M.R. (2011). Teach the Children Well: A Holistic Approach to Developing Psychosocial and Behavioral Competencies Through Physical Education. Quest.

[31] Hardman K., Murphy C., Routen A., Tones S. (2014). World-Wide Survey of School Physical Education: Final Report.

[32] Rozporządzenie Ministra Edukacji Narodowej z Dnia 30 Stycznia 2018 r. w Sprawie Podstawy Programowej Kształcenia Ogólnego Dla Liceum Ogólnokształcącego, Technikum Oraz Branżowej Szkoły II Stopnia. http://isap.sejm.gov.pl/isap.nsf/DocDetails.xsp?id=WDU20180000467.

[33] Osiński W. (2011). Teoria Wychowania Fizycznego (Theory of Physical Education).

[34] Sutherland S., Legge M. (2016). The Possibilities of “Doing” Outdoor and/or Adventure Education in Physical Education/Teacher Education. J. Teach. Phys. Educ..

[35] Asensio-Ramon J., Álvarez-Hernández J.F., Aguilar-Parra J.M., Trigueros R., Manzano-León A., Fernandez-Campoy J.M., Fernández-Jiménez C. (2020). The Influence of the Scout Movement as a Free Time Option on Improving Academic Performance, Self-Esteem and Social Skills in Adolescents. Int. J. Environ. Res. Public. Health.

[36] Priest S., Gass M. (2017). Effective Leadership in Adventure Programming, 3E.

[37] Hattie J., Marsh H.W., Neill J.T., Richards G.E. (2016). Adventure Education and Outward Bound: Out-of-Class Experiences That Make a Lasting Difference. Rev. Educ. Res..

[38] Leśny A., Lisin I. (2018). Outdoor Academy Manual of Educational Program for Youth Groups.

[39] Martin A.P.P., McCullagh D.J. (2011). Physical Education & Outdoor Education: Complementary but Discrete Disciplines. Asia-Pac. J. Health Sport Phys. Educ..

[40] Czyż M., Nowakowska M. (2014). Zdobywamy “Koronę Gór Polski”. Projekt autorskiego programu nauczania wychowania fizycznego w ramach dwóch obowiązkowych godzin zajęć fakultatywnych. Przygoda w Edukacji i Edukacja w Przygodzie. Outdor i Adventure Education w Polsce.

[41] Jędrzejczyk M., Łachacz I. (2015). Outdoor- przyjaciel procesu kształcenia. Outdoor-Owy Zawrót Głowy.

[42] Bull F.C., Al-Ansari S.S., Biddle S., Borodulin K., Buman M.P., Cardon G., Carty C., Chaput J.-P., Chastin S., Chou R. (2020). World Health Organization 2020 Guidelines on Physical Activity and Sedentary Behaviour. Br. J. Sports Med..

[43] Dzwonkowska I., Lachowicz-Tabaczek K., Łaguna M. (2008). Samoocena i Jej Pomiar. Polska Adaptacja Skali SES M. Rosenberga. Podręcznik.

[44] Prochaska J.J., Sallis J.F., Long B. (2001). A Physical Activity Screening Measure for Use With Adolescents in Primary Care. Arch. Pediatr. Adolesc. Med..

[45] Małkowska-Szkutnik A., Mazur J. (2011). Wyniki Badań HBSC 2010: Raport Techniczny.

[46] McKenzie M. (2000). How Are Adventure Education Program Outcomes Achieved?: A Review of the Literature. Aust. J. Outdoor Educ..

[47] Conrad D., Hedin D. (2016). National Assessment of Experiential Education: Summary and Implications. J. Exp. Educ..

[48] Barton J., Bragg R., Pretty J., Roberts J., Wood C. (2016). The Wilderness Expedition: An Effective Life Course Intervention to Improve Young People’s Well-Being and Connectedness to Nature. J. Exp. Educ..

[49] Neill J.T., Richards G.E. (1998). Does Outdoor Education Really Work? A Summary Of Recent Meta-Analyses. J. Outdoor Environ. Educ..

[50] Catalano R.F., Berglund M.L., Ryan J.A.M., Lonczak H.S., Hawkins J.D. (2002). Positive Youth Development in the United States: Research Findings on Evaluations of Positive Youth Development Programs. Prev. Treat..

[51] Agans J.P., Maley M., Rainone N., Cope M., Turner A., Eckenrode J., Pillemer K. (2020). Evaluating the Evidence for Youth Outcomes in 4-H: A Scoping Review. Child. Youth Serv. Rev..

