# Supplementary material for: The Impact of Physical Education Based on the Adventure Education Programme on Self-Esteem and Social Competences of Adolescent Boys
Source: Int J Environ Res Public Health. 2021 Mar 15;18(6):3021. doi: 10.3390/ijerph18063021 (PMC8000378; doi:10.3390/ijerph18063021)
Supplement: Supplementary file 1 [file ijerph-18-03021-s001.pdf]

Supplementary Material

Overview of the Programme

Task related to Adventure Education (The amount of classes)	Task course	Competencies used over the course of tasks	Task goals
Openning of the project. Participants get to know each other. (2)	Presentation of the programme goals and structure. Discussion of expectations and concerns as well as establishing common rules.	social exposure, self-awareness and self-esteem building	getting the participants to know each other, creating an atmosphere of trust and safety
Joint problem solving tasks.(16)	The course of the task in accordance with the stages of learning by doing: presentation of the task instructions, time for the participants for group planning of task execution and the division of roles, an attempt to perform the task joint reflection performing, the task again considering the reflection The trainer's role is to make sure that the safety rules are adhered to and to moderate the reflection process.	cooperation to achieve joint group goal, communication, assertiveness, social exposure, negotiations, action strategy planning	perfecting social competencies, building self-awareness and self-esteem
Tasks building mutual trust and self trust.(4)	The required level of trust during task participation increases with each subsequent exercise.	deeper confidence in one's abilities building trust to other participants, empathy, communication, assertiveness, cooperation	perfecting social competencies, building self-awareness and self-esteem
Closing of the programme, the final reflection.(2)	Reviewing all the classes with the participants in the form of presentations including photos and video. Reflection using the attributes that appear throughout the entire programme (e.g. blindfolds, boxes, ropes, hula hoops, etc.)	social exposure, self-awareness and self-esteem building	involving participants in the process of reflection, analysis of changes on the individual and group level, closing the project
